# Structural Insight Into a Human H Ferritin@Gold‐Monocarbene Adduct: Aurophilicity Revealed in a Biological Context

**DOI:** 10.1002/anie.202503778

**Published:** 2025-04-27

**Authors:** Lucrezia Cosottini, Andrea Giachetti, Annalisa Guerri, Ane Martinez‐Castillo, Andrea Geri, Stefano Zineddu, Nicola G. A. Abrescia, Luigi Messori, Paola Turano, Antonio Rosato

**Affiliations:** ^1^ Department of Chemistry “Ugo Schiff” University of Florence Via della Lastruccia 3–13 Sesto Fiorentino 50019 Italy; ^2^ Consorzio Interuniversitario Risonanze Magnetiche di Metallo Proteine (CIRMMP) Sesto Fiorentino FI 50019 Italy; ^3^ Structure and Cell Biology of Viruses Lab Center for Cooperative Research in Biosciences (CIC bioGUNE) Basque Research and Technology Alliance (BRTA) Derio Spain; ^4^ IKERBASQUE, Basque Foundation for Science Bilbao Spain; ^5^ Magnetic Resonance Center (CERM) University of Florence Via Luigi Sacconi 6 Sesto Fiorentino FI 50019 Italy

**Keywords:** Aurophilic interactions, Cryo‐EM, Ferritin, Gold(I), Metallo‐drugs

## Abstract

Human H ferritin (HuHf) has excellent potential as a nanocarrier for the selective delivery of anticancer metal‐based drugs to tumor cells. Here, we addressed the interaction of the gold monocarbene compound Au(NHC)Cl with HuHf by electrospray ionization‐mass spectrometry (ESI‐MS) measurements, which provide the metalation state of the protein subunits and demonstrate the involvement of protein cysteines in gold binding. The adduct between Au(NHC)Cl and HuHf was studied by cryo‐EM measurements, resulting in a high‐resolution 3D density map at 1.51 Å. The cryo‐EM structure shows a novel tetranuclear gold(I) cluster, located in a surface pocket of each subunit where it is bound to Cys90 and Cys102. The short inter‐metal distances are diagnostic of the occurrence of aurophilic interactions. The present work demonstrates the usefulness of cryo‐EM to investigate the interactions between metal‐based drugs and their protein targets/carriers, also leveraging the strong signal of transition metal ions.

## Graphical abstract

Human H ferritin (HuHf) is a nanocarrier for anticancer metal drugs. By cryo‐EM, a 1.51 Å structure of its adduct with Au(NHC)Cl was solved, showing a novel tetranuclear gold(I) cluster bound to Cys90 and Cys102. Short inter‐metal distances indicate aurophilic interactions. This study highlights the power of cryo‐EM in examining metal‐drug–protein interactions using the strong signal of transition metal ions.

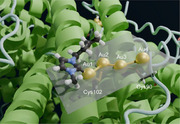

## Introduction

Drug nanocarriers function by protecting the drug from premature degradation, inhibiting early interaction of the drug with the biological environment, enhancing cellular penetration, and controlling pharmacokinetic and distribution profiles via passive or active cell targeting.^[^ ^1^ ^]^ Active delivery to specific cell types involves the functionalization of the nanocarrier surface with targeting moieties (e.g., peptides or receptor ligands) so that the carrier is internalized into cells prior to drug release. The most common drug delivery systems are lipid‐based materials (e.g., liposomes and micelles), polymers (e.g., polyethylene glycol, poly(lactic‐co‐glycolic acid), dendrimers), carbon‐based materials (e.g., fullerenes and nanotubes, including quantum dots), and inorganic nanoparticles (e.g., iron oxide or gold nanoparticles). Despite their chemical diversity, they all fall within an ideal size range of 5–200 nm. In addition, viruses and protein cages can be considered.^[^ ^2^ ^]^ A typical example is provided by mammalian ferritin, which possesses an essentially spherical architecture deriving from the self‐assembly of 24 protein protomers. The resulting structure, which is extremely stable over a wide range of temperatures and pHs, has an external diameter of about 12‐nm and an 8‐nm internal cavity.

The use of ferritin as a carrier for drugs and/or imaging probes has been tested largely using the commercially available horse spleen protein.^[^ ^3^ ^]^ More recently, the focus has shifted toward recombinant human ferritins because of their low immunogenicity,^[^ ^4^ ^]^ reduced toxicity and side effects, and high dose tolerance.^[^ ^5^ ^]^ Additionally, ferritin is reported to cross the blood–brain barrier.^[^ ^6^ ^]^ In nature, cytoplasmic ferritins are heteropolymers resulting from the combination of two types of protomers: the H chain (heavy chain, 21 kDa) and the L chain (light chain, 20 kDa),^[^ ^7^ ^]^ in ratios that depend on their tissue/organ localization or health status. Recombinant ferritins can be produced in vitro as homopolymers (fully H or fully L in composition). The former (HuHf, hereafter) targets human cells through transferrin receptor 1 (TfR1), which is expressed in high levels in several malignancies^[^ ^8^ ^]^; the latter (HuLf) has been proposed to target Scavenger Receptor Class A member 5 receptor (Scara5),^[^ ^9^ ^]^ although this interaction remains less defined and characterized. The simultaneous binding and uptake of HuHf was demonstrated, highlighting how the protein is able to dissociate from the TfR1 in the endosomes and translocate to lysosomes.^[^ ^10^ ^]^

Thanks to its internal cavity, ferritin can host various inorganic and organic materials. Such materials can be incorporated via the so‐called C3 channels, which connect the cavity with the external bulk solution, or by exploiting the ability of ferritin to reversibly disassemble and self‐reassemble.^[^ ^3^ ^]^ The internalization of small molecules that fit the size of the channels through passive diffusion has been widely used with inorganic and organic complexes.^[^ ^11^, ^12^, ^13^, ^14^ ^]^ Additionally, the large external surface of ferritin is suitable for modification thanks to the well‐defined structure of HuHf and its stability. Different modifications for various approaches have been performed over the years. Reactions with N,N‐dimethyl 1,3‐diaminopropane^[^ ^15^ ^]^ produced a “cationic ferritin” for transmission electron microscopy; the modified cationic ferritins have been reacted with anionic polymer surfactant and poly(ethylene glycol) 4‐nonylphenyl 3‐sulfopropyl ether.^[^ ^16^, ^17^ ^]^ Long alkyl chains were attached to ferritin to obtain a fully organic‐soluble hydrophobic particle.^[^ ^18^ ^]^ Also, the modification of ferritin with short polyethylene glycol (PEG) chains has been exploited.^[^ ^19^, ^20^ ^]^

Metal‐based drugs, particularly platinum‐based drugs, are an important class of anticancer drugs in widespread clinical use. However, they usually suffer from severe side effects, most likely due to their broad and poorly selective reactivity, which often leads to treatment failure. This makes the targeting of metallo‐drugs to cancerous tissues even more important than for traditional organic drugs. Consequently, there have been many attempts to prepare and characterize adducts between platinum drugs and a variety of carriers in order to increase the efficacy of delivery.^[^ ^21^ ^]^ Ferritin can be an excellent carrier for metal‐based drugs. Some previous studies have investigated the formation and biological evaluation of ferritin adducts with platinum drugs.^[^ ^22^ ^]^ More recently, we have investigated ferritin as a potential nanocarrier for the gold‐based drugs auranofin and aurothiomalate.^[^ ^23^, ^24^ ^]^ In both cases, biophysical methods provided evidence of the formation of well‐defined derivatives. These bioconjugates have potent antitumor effects in vitro, most likely mediated by their recognition by the TfR1 receptor.^[^ ^8^ ^]^ Here, we have considered a gold(I) monocarbene complex prepared in our laboratories, which has important cytotoxic effects based on a fundamentally different mode of action from that of auranofin.^[^ ^25^ ^]^ Au(NHC)Cl is a cytotoxic gold(I) carbene complex with a 1‐butyl‐3‐methyl‐imidazole‐2‐ylidene moiety as the NHC ligand (where NHC stands for *N‐hetetocyclic carbene*), coordinated to the gold center by a direct gold‐carbon bond; the second gold ligand is a chloride. Au(NHC)Cl acts as a classical prodrug and is activated by the release of the chloride ligand. The resulting Au(NHC)^+^ cation is indeed the truly reactive species, capable of binding tightly to biomolecules by forming strong coordinative bonds, preferentially to free thiol groups, as here demonstrated via ESI‐MS. Through cryo‐EM measurements, we could then observe the formation of a gold nanocluster of unprecedented structure bound to the protein surface.

## Results and Discussion

### Reaction Between Au(NHC)Cl and HuHf Indicates Reproducible Multi‐Metal Binding

**Figure 1:**
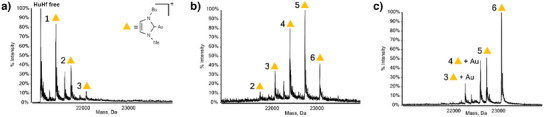
Titration of HuHf with increasing amounts of Au(NHC)Cl: ESI‐MS spectra recorded after incubation with increasing amounts of Au(NHC)Cl: the added Au(NHC)Cl per ferritin subunit ratios are 1, 5 and 8 (a–c). The number of Au(NHC)^+^ ions bound to HuHf subunits are indicated by 1–6. Attempts to go beyond the ratio represented in the spectrum of panel (c) led to massive protein precipitation.

ESI‐MS was used to study the reaction of human ferritin with Au(NHC)Cl. Intact apo‐HuHf was dissolved in phosphate‐buffered saline (PBS) and then reacted, by simple incubation, with increasing amounts of Au(NHC)Cl to obtain HuHf@Au(NHC) adducts. After 3 h of incubation, a variety of adducts with different stoichiometries were formed, depending on the molar ratio of gold to protein subunit used (Figure 1). The identification of gold as the unique metal binding the protein was directly obtained from the ESI‐MS data and was further validated by inductively coupled plasma optical emission spectroscopy (ICP‐OES). The combination of these two analytical techniques is generally exploitable to prove the identity of metal ions in cryo‐EM structures because the same solution is used for metal analysis and for vitrification.

Each subunit of HuHf can bind an increasing number of Au(NHC)^+^ fragments (ranging from 1 to 6) when exposed to increasing concentrations of the metallodrug, with the molar fraction of the adducts carrying a higher number of gold fragments becoming progressively larger. The comparison between the theoretical and experimental masses of the formed adducts is given in Table S1. For all conditions tested, the maximum number of bound fragments was 6. Simulations of the ESI‐MS data indicate that gold remains in the +1 oxidation state upon binding (Figure S1). Since the Au(NHC)^+^ fragment is known to selectively bind protein‐free cysteines with a typical 2:1 metal fragments to cysteine molar ratio^[^ ^26^ ^]^ and a single HuHf subunit contains three free cysteines, we can assume that Au(NHC)^+^ selectively binds the three cysteine residues of monomeric ferritin. Complete and simultaneous binding of 2 Au(NHC)^+^ moieties at all three potential binding sites of all HuHf subunits was never achieved under the ESI‐MS conditions.

To support the hypotheses above, additional ESI‐MS studies were performed on ferritin mutants, namely, C130A, C90AC102A, and C90A, in which some of the three cysteine residues were selectively removed. Their ESI‐MS spectra obtained after incubation with Au(NHC)Cl are shown in Figure S2. The results nicely confirmed our hypothesis. Indeed, in the two cases where a single cysteine residue was removed, a maximum stoichiometry of 4:1 was obtained; when two cysteines were mutated simultaneously, the maximum stoichiometry was 2:1. To further demonstrate that Au(NHC)^+^ fragments selectively bind the three free cysteines, trypsinization experiments were performed on the native protein before and after metalation. The analysis of the tryptic digest identified unambiguously the two cysteine‐containing fragments (87–108 and 125–143) as being involved in metal binding (Figure S3).

### Tetra‐Gold Cluster is Present at the Surface of the HuHf Nanocage

The best cryo‐EM grids were obtained by vitrification of samples prepared with the Au(NHC)Cl:protein ratio corresponding to the middle panel of Figure 1. Using a total number of 2 736 819 particle images, we obtained a map at 1.51 Å resolution as shown by the gold‐standard Fourier shell correlation (FSC) criteria (Figure S4), which enabled us to build the 3D structure of the HuHf@Au(NHC) adduct.

**Figure 2:**
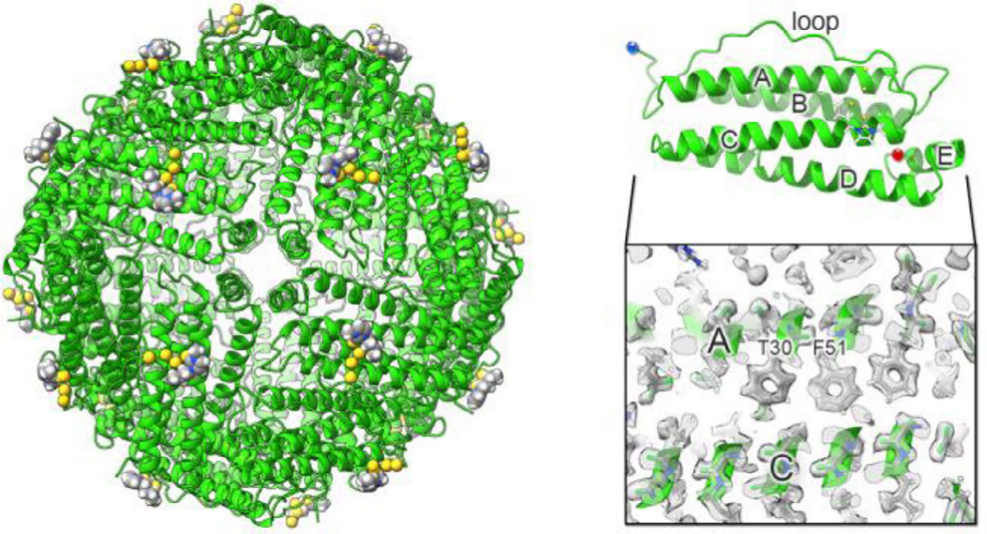
The HuHf nanocage. The overall structure of HuHf is depicted as a green cartoon with the tetranuclear gold cluster (in yellow) and the NHC moiety (C in grey, H in white and N in blue) bound to the external surface of each subunit shown in spacefill representation. On the right side of the figure, the secondary structure of a single subunit is shown, with the labeling of the helices (A to E) that will be used throughout the paper. The inset at the bottom displays the density at helices A and C, with selected side chains shown to illustrate the quality of the density. The blue and red spheres indicated the N‐ and C‐termini of the protomer, respectively.

Some quality parameters are given in Table S2, showing that the obtained structure is of very good quality. The octahedral symmetry of HuHf was preserved also in the adduct and each protomer closely maintained the backbone conformation observed in previous X‐ray and cryo‐EM structures of apo ferritin (Figure 2).

In fact, the backbone root mean square deviation (RMSD) of a single subunit to the 1.25 Å cryo‐EM structure of human apoferritin (PDB code 6Z6U) was 0.102 Å (over 154 residues), whereas the RMSD to the 1.34 Å X‐ray structure of iron‐loaded HuHf (PDB code 4Y08) was 0.160 Å (over 162 residues). With the achieved resolution, it was possible to identify alternative side chain conformations, such as Arg59, as well as to observe the densities of hydrogens in the most ordered parts of the structure (Figure 2), as previously reported for atomic‐resolution cryo‐EM structures.^[^ ^27^ ^]^ After fitting the polypeptide chain in the map, we analyzed the presence of possible metal ions in the structure. At the ferroxidase site the only observable density was interpretable as due to the presence of multiple water molecules, based on the hydrogen bond patterns and the intensity of the density itself, at variance with what reported in other ferritin structures.^[^ ^27^, ^28^ ^]^ Nevertheless, the configuration of the side chains of the protein residues forming the site is very close for all structures, except where an iron cluster is bound.^[^ ^29^ ^]^ Density suggestive of the occurrence of a water molecule or a non‐transition metal ion is present at the three‐fold axis channel (Asp131/Glu134), which is the hydrophilic channel that allows cations to move from the bulk solution to the inner cavity.^[^ ^30^, ^31^ ^]^

**Figure 3:**
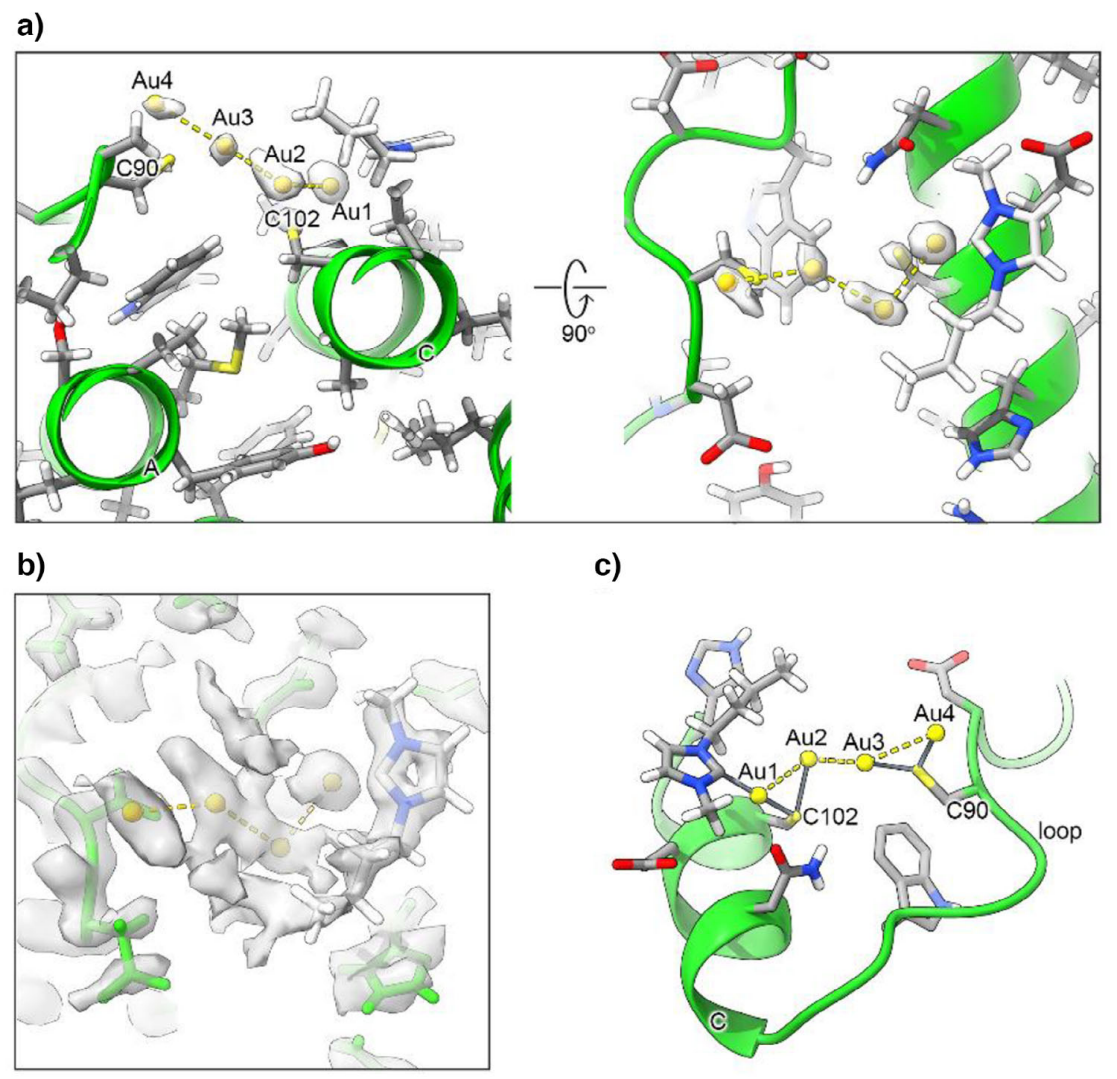
Cryo‐EM density map of bound ligands. a) Density in transparent white showing the vicinity of the two solvent‐exposed Cys residues (Cys90 and Cys102, depicted as sticks) fitted with the tetranuclear gold cluster. Gold atoms are labeled Au from 1 to 4, and the labels A and C mark the ferritin helices. The density, gaussian filtered (see Materials and Methods) was displayed in ChimeraX^[^ ^32^ ^]^ at a contour level of 2.7 σ; b) the same region as the right view in a), but with the carbene moiety fitted into the original density displayed at 2.46 σ; c) different view of the complexation region with the coordination bonds between the gold(I) ions, and the cysteine and NHC moieties displayed in grey stick. Helix C contains Cys102, whereas Cys90 is part of the BC loop.

The ESI‐MS data described in the previous section prompted us to inspect the environment around each of the three cysteine residues. While Cys130 did not show prominent features, a very significant density was observed in the proximity to Cys90 and Cys102, which are both solvent‐exposed. The intensity in this region was somewhat stronger than even for the surrounding atoms of the protein backbone (Figure 3).

Based on the shape of the density, we fitted it with dummy metal ions, i.e., with null van der Waals (VDW) radius, in a number ranging from one to four. This strategy was adopted to determine the number of ions to be fitted in the density without any prejudice. At the same time, neglecting VDW interactions allowed the metal‐protein as well metal‐metal distances to become short enough to indicate the occurrence of a coordination bond or of a metal‐metal interaction. Indeed, in separate calculations run with the correct VDW radius for the gold(I) ions the metals were pushed far away from each other and from the possible donor atoms from the protein, which resulted in a poorer fit to the local density (not shown). With this procedure, we estimated the presence of four metal ions in the surface pocket (Figure 3a).

**Figure 4:**
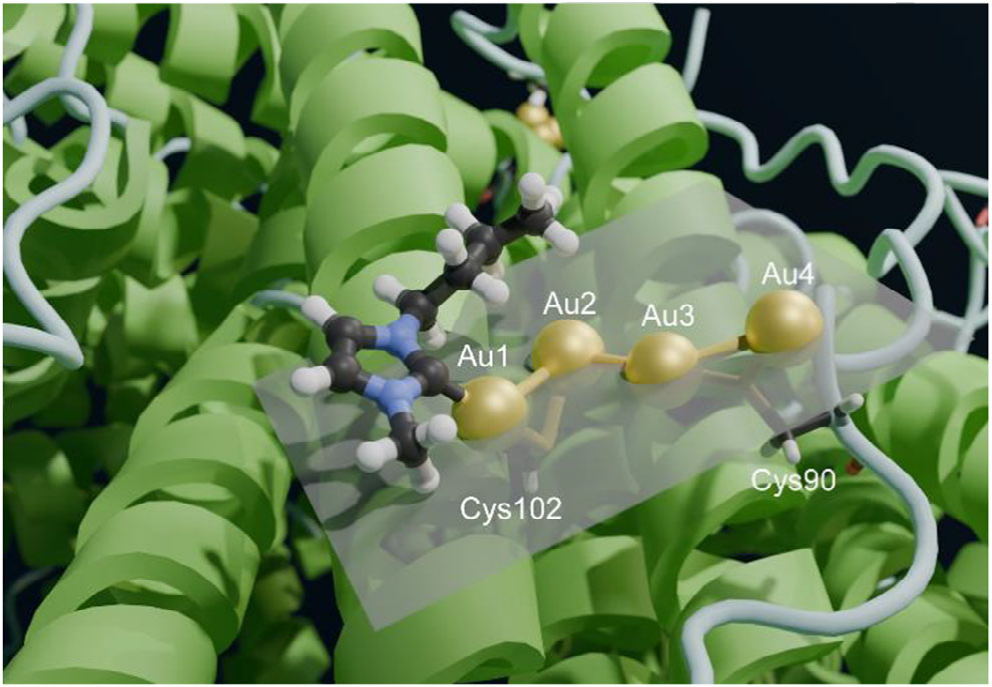
Structure of the tetranuclear gold cluster. The gold ions are represented by gold spheres. Gold sticks indicate the proposed inter‐gold aurophilic interactions, as well as the coordinative bonds to the Sγ atoms of the Cys90 and Cys102 residues. The near‐planarity of the tetragold cluster is highlighted by the average plane of the four metal ions. Both Cys ligands are below the plane.

A similar approach was implemented to position the carbene moieties in the cavity. In this case, visual inspection of the density highlighted only one carbene ring with clarity (Figure 3b). Consequently, we preferred to build a structure with the four gold(I) ions and a single carbene moiety (Figure 3c). The pocket in which the Au(NHC)^+^ fragment lies is quite shallow; it is defined by the space between helices A and C, and the BC loop (Figure 4). In the proximity of Cys102, its surface wraps the plane and butyl chain of the carbene ligand.

We deposited this structure with PDB code 9HQ6 and EMDB code EMD‐52339. The residual density in the proximity of the gold(I) ions suggested the possible position for at least two, possibly three, additional donor atoms to the metals, which we hypothesized to be the carbon C(2) atoms of as many additional carbene moieties. However, weak density was observed indicating that the rings of the latter experience conformational heterogeneity. Thus, although a 3D structural model with four carbene molecules bound to the gold(I) ions can be built, upon introduction of the appropriate carbon‐gold distance restraints, we regarded it as more of a computational model. Nevertheless, this model showed that the pocket has indeed sufficient space to host four gold(I) ions together with four carbene groups, without compromising the agreement of the polypeptide conformation with the experimental density.

The structure clearly shows that the two sulfur atoms of the Cys90 and Cys102 side chains coordinate to two gold(I) ions each (Figures 3c and 4), at a distance of 2.2–2.3 Å, with the exception of the distance between the Sγ of Cys102 and the second gold(I) ion (Au2), which was 2.5 Å (Table 1). The coordination distance between C(2) of the carbene ring and the first gold(I) ion (Au1) was 2.1 Å. The gold(I) ions formed a zig‐zag chain (Figure 4), with the distance between two consecutive gold(I) ions being 3.1–3.2 Å. Very similar coordination as well as metal‐metal distances were reported in previous work,^[^ ^33^, ^34^, ^35^ ^]^ although the present arrangement of the metal ions is novel. A possible description of the present configuration is that each Cys residue coordinates a pair of gold(I) ions, and the two pairs interact via a contact between two of their ions at about the same distance (Figure 3c). Notably, the arrangement of the four ions is quite close to planarity (Figure 4), as shown by a dihedral angle of 198°. For this analysis, we also note that the density of Au2 has a more distorted shape than the others, possibly suggesting that this ion can occupy at least two slightly different positions within the cluster (Figure 3a). We compared these geometrical parameters with the distances computed for a cysteine‐gold(I)‐carbene adduct using density functional calculations,^[^ ^24^ ^]^ and found the distances observed here for Au1 to be in very good agreement with the theoretical values.

**Table 1:** List of distances, angles, and dihedrals of the cryo‐EM structure of HuHf@Au(NHC).

**Distance (Å)**	
Au1–Au2	3.1
Au2–Au3	3.0
Au3–Au4	3.2
C(2)–Au1	2.1
Sγ 102–Au1	2.2
Sγ 102–Au2	2.5
Sγ 90–Au3	2.3
Sγ 90–Au4	2.2
**Angle (°)**	
C(2)–Au1–Sγ102	163
Au1–Sγ102–Au2	81
Au3–Sγ90–Au4	87
Au1–Au2–Au3	94
Au2–Au3–Au4	137
C(2)–Au1–Au2	125
**Dihedral (°)**	
Au1–Au2–Au3–Au4	198
Sγ102–Au2–Au3–Sγ90	100

As mentioned, the observed density allows only one carbene to be confidently positioned, although the ESI‐MS data suggest that each gold(I) ion binds one carbene moiety. We hypothesize that this is because the three undetectable carbene rings are quite mobile and therefore their density is too weak to be observed. Based on the 3D structure, the reason for the Au1‐bound carbene ring being much more ordered than the others could be that it is making contacts to the side chains of residues in the N‐terminal part of helix C, which restricts its available conformational space. Instead, the other rings can be modeled as protruding into the bulk solution, leaving them with greater freedom.

The electrostatic potential at the surface of the pocket is negative, especially in the proximity of the gold(I) ions, while it is close to neutral in the proximity of the butyl chain in position 1 of the ring (Figure S5).

No additional density associated with the possible presence of other gold atoms bound to the protein was detected. In particular, a detailed examination of the local environment of Cys130 ruled out the presence of any bound gold atom, at variance with what was observed by ESI‐MS in the disassembled cage (Figure 1b). In the native structure, Cys130 is the least accessible of the three cysteines with a fractional accessible surface area of only 10% with respect to 26%–36% for Cys102 and Cys90, respectively. We can interpret the ESI‐MS results by assuming that Au(NHC)^+^ cations can transit through the C3 channels, where Cys130 is located, without forming a stable complex in the assembled cage, eventually reaching the inner cavity. Under the ESI‐MS conditions, the cage is disassembled into monomers, thereby releasing its cargo, which would then bind to the now‐exposed Cys130.

Our 3D structure revealed the presence of a well‐defined string of four close gold atoms with relative distances at 3.1–3.2 Å. We hypothesize that these short inter‐metallic distances can be explained by the occurrence of significant aurophilic interactions. The term “aurophilicity” was first introduced in 1989 to describe phenomena in the structural chemistry of gold that could not be readily interpreted by the conventional concepts of chemical bonding.^[^ ^36^, ^37^ ^]^ In the absence of steric hindrance, gold(I) centers, either within a given molecule or in different molecules, can be observed at equilibrium distances in the range 2.50–3.50 Å, well below the sum of their VDW radii (3.80 Å). Such attractive interactions were unexpected since gold(I) centers have a [5d^10^] closed‐shell electronic configuration and thus should experience only weak VDW forces. Furthermore, the interactions between the (+1) electrical charges of the gold cations or between gold atoms with the same polarization in their covalent bonds should result in significant Coulomb repulsion at any shorter distance. Such weak metallophilic contacts established between gold(I) ions are now referred to as aurophilic interactions; they are attractive interactions between linearly two‐coordinate gold ions atoms in the +1 oxidation state. Thanks to the fundamental contributions of Hubert Schmidbaur,^[^ ^35^, ^37^ ^]^ the concept of the aurophilic interaction is now well established, and the complex theoretical aspects associated with aurophilicity have been elucidated as well.^[^ ^36^, ^37^ ^]^

In this work, we highlighted the excellent capability of cryo‐EM to provide high‐resolution 3D structures of drug‐cage adducts. Furthermore, we demonstrated that wild‐type ferritin may act as a scaffold, assisting in the formation of soft metal clusters with defined stoichiometry on its surface rather than within its inner cavity, thanks to the presence of solvent‐exposed cysteine residues.^[^ ^38^, ^39^ ^]^ Multinuclear iron clusters assemble with relative ease in the inner cavity of ferritin, as this is intrinsic to its biochemical function, namely, the formation of hydrated ferric oxide biominerals within the cavity; the initial phases of the biomineralization process are mediated by carboxylate side chains.^[^ ^29^, ^40^, ^41^ ^]^ Other metals besides iron can form biominerals in the cavity.^[^ ^42^ ^]^ As far as noble metals are concerned, an engineered variant of HuHf with a total of 96 cysteines and histidines removed from the exterior surface and 96 non‐native cysteines added to the interior surface promoted the formation of silver or gold nanoparticles within the protein cavity.^[^ ^43^ ^]^ In addition, gold nanoparticles can be built by reducing Au(III)‐containing anions, such as AuCl_4_^−^, previously loaded inside the cavity of horse heart ferritin (which is largely made by L subunits that do not contain any surface Cys).^[^ ^44^ ^]^ This approach can be further facilitated by introducing new Cys residues via site‐directed mutagenesis in L‐ferritin, thus increasing the affinity of the cavity for gold ions, leading to the formation of sub‐nanoclusters containing up to 10 metal ions.^[^ ^34^ ^]^ In these complexes, various linear units are present, with gold(I) ions coordinated by two Cys or by one Cys and one His. More recently, the formation of multinuclear gold(I) clusters with 8–12 ions was accomplished with a suitably designed L‐ferritin variant incubated with a large excess (50–400 equivalents) of chloro(dimethylsulfide) gold(I).^[^ ^38^ ^]^ In all these systems, various stoichiometries were observed; in addition, binding of one or two gold(I) ions at Cys residues exposed on the inner cavity surface also occurred, often with partial occupancy.^[^ ^38^ ^]^

Instead, cysteine residues present on the external surface of wild‐type HuHf are able to capture gold(I) compounds. This has been reported for auranofin, where only a minor fraction of the added gold was encapsulated in the inner cavity via disassembly/reassembly of the cage, while the remaining ions formed disordered gold clusters at Cys90 and Cys102.^[^ ^39^ ^]^ When auranofin is added by simple incubation, it only binds to the solvent‐exposed cysteines.^[^ ^23^ ^]^ A similar situation was modeled for aurothiomalate.^[^ ^24^ ^]^

In this context, the present investigation stands out because we obtained and structurally characterized a well‐defined four‐ion cluster that also included one of the exogenous carbene ligands. Three other ligands were probably present but too mobile to be observed in the cryo‐EM maps. With our setup, the cluster formed spontaneously and reproducibly at the surface pocket that is lined up by Cys90 and Cys102. Additional Au(NHC)^+^ ions presumably entered the inner cavity but were not observable, besides via ESI‐MS at the Cys130 after disassembling the cage. The observed order in the structure results from the match among the stereochemical features of the carbene ligand, the spatial features of the surface pocket, and the disposition of the protein atoms that define it. In turn, this enables the energetically favorable 3D arrangement of the gold(I) ions.

## Conclusion

Our work shows that the formation of well‐defined, ordered gold(I) clusters in wild‐type ferritin is feasible with a relatively straightforward approach. This is presumably due to the good complementarity between the stereochemical properties of the ligand in the Au(NHC)^+^ ion and of the geometry and reactivity of the surface pocket identified in the HuHf structure that contains the Cys90‐Cys102 dyad. This observation can be exploited for the design of novel molecules containing soft metals that target the same pocket. From an inorganic chemistry perspective, the present 3D structure offers an excellent and unprecedented example of the occurrence of an aurophillic interaction within a biological system and underlines its importance. Indeed, the chain of four closely spaced gold(I) ions observed here is stabilized not only by their coordinative bonds to the sulfur atoms of Cys90 and Cys102, but also by the relatively strong gold‐gold metallophilic interactions. Similar interactions have previously been characterized at a comparable resolution only in chemically synthesized clusters or in designed ferritin variants.^[^ ^38^ ^]^ Thus, the present study highlights the biological significance of aurophilic interactions for gold‐based drugs targeting a native protein.

More generally, this study demonstrates the potential of the cryo‐EM approach to identify and characterize heavy metal binding sites on proteins at high resolution due to the stronger density resulting from the presence of the metal ion(s). This is particularly important in the context of interactions between metal‐based drugs and the proteins that represent their biological target or are used as their nanocarriers. In fact, most of these inorganic drugs contain metal ions of the second and third transition series (e.g., Ru, Ag, Ir, Pt, Hg), which are expected to behave similarly to Au.

## Supporting Information

The authors have cited additional references within the Supporting Information. ^[^ ^23^, ^32^, ^40^, ^45^, ^46^ ^]^

## Conflict of Interests

The authors declare no conflict of interest.

## Supporting information



Supporting Information

## Data Availability

The 3D structure and cryo‐EM map were deposited, respectively, in the Protein Data Bank (PDB ID: 9HQ6) and the Electron Microscopy Data Bank (EMDB ID: EMD‐52339).
