# Supplementary material for: Structural Insight Into a Human H Ferritin@Gold‐Monocarbene Adduct: Aurophilicity Revealed in a Biological Context
Source: Angew Chem Int Ed Engl. 2025 Apr 27;64(30):e202503778. doi: 10.1002/anie.202503778 (PMC12281091; doi:10.1002/anie.202503778)
Supplement: Supplementary file 1 — Supporting Information [file ANIE-64-e202503778-s001.docx]

Structural Insight into a Human H Ferritin@Gold-Monocarbene Adduct: Aurophilicity Revealed in a Biological Context

Lucrezia Cosottini,^[a]^ Andrea Giachetti,^[b]^ Annalisa Guerri,^[a]^ Ane Martinez-Castillo,^[c]^ Andrea Geri,^[a]^ Stefano Zineddu,^[a]^ Nicola G.A. Abrescia,^[c,d]^ Luigi Messori,^[a]^ Paola Turano,^[a,e]^ Antonio Rosato, * ^[a,e]^

[a] L. Cosottini, Dr. A. Guerri, A. Geri, S. Zineddu, Dr. L. Messori,

Department of Chemistry “Ugo Schiff”

University of Florence

Via della Lastruccia 3-13, 50019 Sesto Fiorentino, Italy

[b] Dr. A. Giachetti

Consorzio Interuniversitario Risonanze Magnetiche di Metallo Proteine (CIRMMP)

50019, Sesto Fiorentino, FI, Italy

[c] A. Martinez-Castillo

Structure and Cell Biology of Viruses Lab, Center for Cooperative Research in Biosciences (CIC bioGUNE) Basque Research and Technology Alliance (BRTA); Derio, Spain

[d] Dr. N. G.A. Abrescia

IKERBASQUE, Basque Foundation for Science

Bilbao, Spain.

[e] Dr. P. Turano, Dr. A. Rosato

Magnetic Resonance Center (CERM)

University of Florence

50019, Via Luigi Sacconi 6, Sesto Fiorentino, FI, Italy

E-mail: [antonio.rosato@unifi.it](mailto:antonio.rosato@unifi.it)

**SUPPLEMENTARY INFORMATION**

***Materials and methods***

**Chemicals**

Au(NHC)Cl is a gold(I) carbene with the 1-butyl-3-methyl-imidazole-2-ylidene moiety acting as the NHC ligand of the gold center through a direct gold-carbon bond; the coordination is completed by a labile chloride. The synthesis of this compound is reported in Messori et al.^[1]^

**Protein production and purification**

Human heavy chain ferritin (HuHf) was expressed in and purified from *E. coli* cells as previously described.^[2]^ Briefly, the pEt-9a plasmid, carrying the HuHf gene, was transformed in BL21(DE3)pLysS competent cells, which were then grown in Luria-Bertani medium until an optical density of 0.6-0.8. To induce the over-expression of the protein, 1 mM IPTG was added and the cells remained under agitation (180 rpm) for 4 h at 37°C. Cells were harvested through centrifugation at 7500 rpm for 15’ at 4°C. The pellet was disrupted by sonication and clarified by ultracentrifugation (40 000 rpm, 40’, 4°C). The supernatant was heated up to 65°C for 15’, to discard the unwanted proteins that are not stable at high temperature, followed by a second ultracentrifugation. The final supernatant was purified with an anionic exchange chromatography with a 0-1.0 M gradient of NaCl, followed by size exclusion chromatography. Finally, the purified ferritin underwent dialysis against 5 L 20 mM TRIS pH 7.5 in the presence of reducing and chelating agents to remove the iron inside the inner cavity to obtain the apo protein. The plasmids for the C130A, C90AC102A and C90A mutants of HuHf were produced in a previous study and expressed following the same protocol as the wild type protein.^[2,3]^

**Preparation of the gold(I) adduct, HuHf@Au(NHC)**

Binding of Au(NHC)^+^ to HuHf was successfully achieved by simple incubation of the Au(NHC)Cl compound with the ferritin nanocage in PBS, using different compound:nanocage ratios, followed by stirring at 37 °C for 3 h and extensive dialysis. This method has the advantage of not requiring any cage disassembly/reassembly. The adducts with the Cys🡪Ala ferritin variants were prepared with a similar approach.

**ESI-MS experiments**

The protein solutions were diluted with 20 mM ammonium acetate solution, pH 6.8, to a final protein concentration of 5x10^-6^ M. 1% v/v of LC-MS grade formic acid was added just before infusion in the mass spectrometer. The ESI mass spectra were acquired through direct infusion at 7 μLmin^-1^ flow rate in a TripleTOF® 5600+ high-resolution mass spectrometer (Sciex, Framingham, MA, U.S.A.), equipped with a DuoSpray® interface operating with an ESI probe. The ESI source parameters were as follows. HuHf: positive polarity, Ionspray Voltage Floating 5500 V, Room Temperature, Ion source Gas 1 (GS1) 40 L/min; Ion source Gas 2 (GS2) 0; Curtain Gas (CUR) 20 L/min, Declustering Poten-tial (DP) 30 V, Collision Energy (CE) 10WT V, acquisition range 650-3300 m/z. The Analyst TF software 1.7.1 (Sciex) was used for acquisition; deconvoluted spectra were obtained by using the Bio Tool Kit micro-application v.2.2 embedded in the Peak-ViewTM software v.2.2 (Sciex). The same protocol was adopted for HuHf, HuHf@Au(NHC) and the corresponding cysteine mutants.

The simulation of the oxidation state of gold was performed using the Peak-ViewTM software v.2.2 (Sciex), where we generated a theoretical spectrum of the adduct assuming gold either in the +1 (Supplementary information Figure 1) or +3 (data not shown) oxidation state. The calculated spectra were then overlaid with our experimental ESI-MS spectra, showing an excellent correspondence in terms of isotopic and mass distribution.

**Trypsin digestion of ferritin and LC-MS analysis:** Ferritin samples in 20 mM ammonium acetate buffer were heated at 100 °C for 5 min and then allowed to cool off at 37 °C. The samples were subsequently incubated for 24 h at 37 °C with a solution of trypsin at a 1:20 trypsin: ferritin subunit ratio. The peptide mixture was characterized using the micro-LC Eksigent Technologies (Eksigent, Dublin, CA, USA) system including a micro LC200 Eksigent pump with flow module 5–50 µL and interfaced with a TripleTOF 5600+ mass spectrometer (Sciex, Framingham, MA, U.S.A.) equipped with DuoSpray Ion Source and Calibrant Delivery System. The stationary phase was a Halo C18 column (0.5 x 100 mm, 2.7 µm; Eksigent Technologies Dublin, CA, USA). The mobile phase was a mixture of 0.1% (v/v) formic acid in water (A) and 0.1% (v/v) formic acid in acetonitrile (B), eluting at a flow rate of 50.0 µL/min with an increasing concentration of solvent B from 2 % to 50 % in 20 min. Injection volume was 2.0 µL and oven temperature was set at 45 °C. For identification and quantification purposes the mass spectrometer analysis was performed using a mass range of 250–2500 Da (TOF scan with an accumulation time of 0.25 s). The ion source parameters in electrospray positive mode were set as follows: curtain gas (CUR) at 25 L/min, Ion source Gas 1 (GS1) at 40 L/min, and Ion source Gas 2 (GS2) at 25 L/min, ion spray voltage floating (ISVF) at 5500 V, source temperature at 150 C, and declustering potential at 10 V. For acquisition, the Analyst TF software 1.7.1 (Sciex) was used and the MS spectra were obtained by using the Bio Tool Kit micro-application v.2.2 embedded in Peak-ViewTM software v.2.2 (Sciex). Theoretical masses were obtained by using the Mass Calculators extension embedded in Peak-ViewTM software v.2.2 (Sciex). The same protocol was adopted for HuHf, HuHf@Au(NHC).

**Sample preparation for cryo-EM and quality control grid assessment**

The HuHf@Au(NHC) adduct obtained in PBS was used at a concentration ranging from 2 to 3 mg/ml for the preparation of the frozen sample, using Quantifoil Au 1.2/1.3 300 mesh grids holey carbon grids. The grids were firstly glow discharged (45 s, 20 mA) in a Pelco EasiGlow system. Then, 3 μl of the adduct solution was deposited on the grids kept in the chamber of a Vitrobot Mark IV (FEI, USA) at 10 °C and 100% humidity. The grids were then blotted for 2.0 s with force 2 and plunge-frozen into the precooled liquid ethane. These grids were used to optimize the sample preparation protocol, as monitored by the acquisition of atlases and movies with a ThermoFisher^TM^ Glacios^TM^ operating at 200 keV with a FEI Falcon 3 electron counting DED at the Florence Center for Electron Nanoscopy (FloCEN) of the University of Florence. Movies were collected at 120,000× magnification using the EPU software package with a pixel size of 0.96 Å/pixel (Supplementary information Table 1). Grids prepared for the collection of high resolution data replicated the above conditions with the exception that the HuHf@Au(NHC) complex was at a concentration of 2.93 mg/ml and the grids were blotted for 3s with blot force 1. The frozen grids were stored in liquid nitrogen until data collection.

**High-resolution data collection**

For high-resolution data collection, images were acquired with a Titan Krios G4i (ThermoFisher Scientific) operated at 300 keV and equipped with a K3 direct electron detector with a Bioquantum imaging filter (Gatan) at the electron Bio-Imaging Centre (eBIC) at the Diamond Light Source (Didcot, UK). Automated data collection was performed using EPU (v3.8.1.7603) at a nominal magnification of x165,000 and the detector in counted super resolution mode, corresponding to a calibrated pixel size of 0.508 Å/pixel at the specimen. The dose rate over vacuum was set to 16.7 e-/pix/s. Movies were collected with a total dose of 50 e^-^/Å^2^ (fractionated in 50 frames) and a defocus range of –0.4 to –1.8 µm (Supplementary information Table 1).

**Data processing**

CryoSPARC v4.4.1 was used to process the dataset. We imported 31,836 movies and applied patch motion correction; the CTF-fit was estimated using patch CTF estimation. Exposures with CTF fits worse than 5 Å were discarded from the dataset, resulting in 88% acceptance. 230 particles were manually picked to create a template for particle picking. Template picker was used to identify particles and 22,458,732 particles were extracted with a box size of 512 pixels. We adjusted the normalized cross correlation (NCC score >0.500) and power threshold (local power in the range -471-1032) by Inspect Particle Picking to improve the visibility of the particles and remove false positives: the selection provided 2,753,306 particles. The particles were then downsampled to a box size of 256 pixels and then two rounds of 2D classification yielded 2,624,176 particles. The particles were extracted at 512 pixels and a 3D homo-refinement was performed using the EMD-13364 cryo-density map as template. The particles were then used for homogeneous refinement against the EMD-13364 volumes with O symmetry imposed, per-particle defocus optimization, per-group CTF parameterization, Ewald sphere correction enabled, and minimize over per-particle scale enabled, thus resulting in a map with 1.64 Å resolution. The reference-based motion correction routine was applied and the above homorefinement procedure was repeated. The final resolution was 1.51 Å as assessed by the gold-standard Fourier Shell Correlation (**Supplementary information Figure 3**).

**3D structure calculation**

For building the protein shell model, the HuHf structure with PDB code 4Y08 was manually placed into the cryo-EM map using Chimera v.1.17.3, then fit using the ‘fit into map’ command. Coot v. 0.9.8.92 was used to adjust the model and place the ligand into the map. The densities around the Cys90 and Cys102 not populated by protein atoms were manually interpreted in terms of gold atoms and their ligands. Non-standard ligand restraints for the carbene molecule were generated using electronic Ligand Builder and Optimization Workbench (eLBOW). Then, real-space refinement against the map was performed using Phenix v. 1.21-5207-000, minimization_global enabled, local_grid_search enabled, and adp refinement enabled. The symmetry operators were identified from the map using the map_symmetry command and applied using apply_ncs to generate a shell with 24 copies of the asymmetric unit. The NCS-expanded shell was then refined again using real-space refinement with adjusted water molecules, minimization_global enabled, local_grid_search enabled, adp refinement enabled, and NCS constraints enabled. BIOMT operators were found using the find_ncs command, and manually placed in the headers of the .pdb file. The deposited map and structural coordinates were obtained applying a B-factor sharpening of -20, whereas the images in this article were obtained from a calculation based on the automatically computed B-factor.

The figures were generated using ChimeraX^[4]^ and the Molecular Nodes plugin^[5]^ for Blender. Protein solvent accessibility was calculated with VADAR.^[6]^ For the display of the map in Figure 3A, we used phenix.map_box to extract a region surrounding the carbene and gold atoms. The resulting map was loaded into ChimeraX,^[4]^ where it was filtered using a Gaussian filter with a standard deviation of 0.25. The map was then boxed around the relevant atoms and displayed at a contour level of 2.7 σ with ChimeraX.


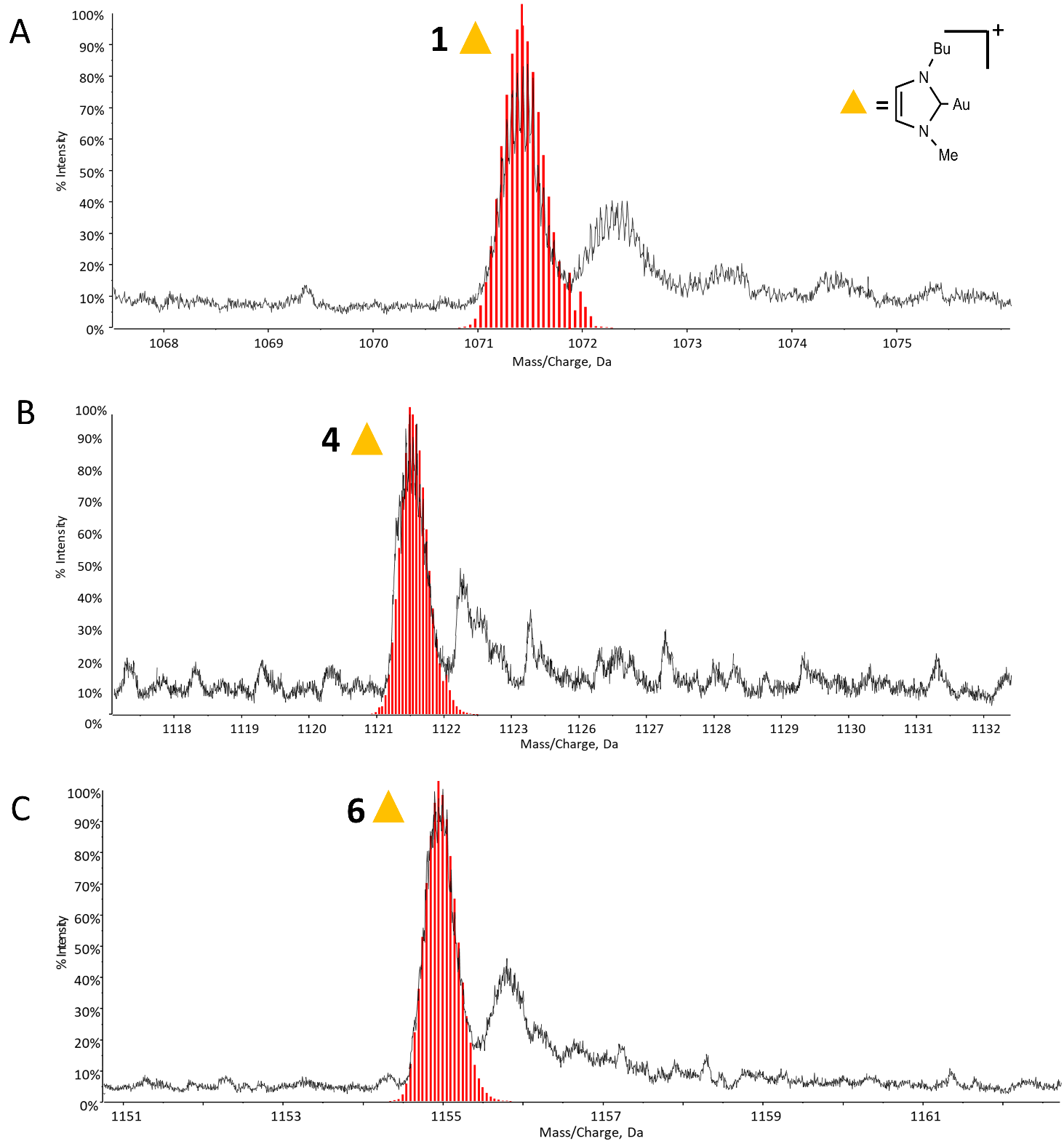


**Supplementary information Figure 1.** Experimental (black) and predicted (red) isotopic patterns of HuHf with 1, 4 and 6 bound Au(NHC)^+^ moieties (A to C); the three panels correspond to the experimental conditions of Figure 1 A, B and C, respectively. The simulation was carried out on the highest intense peak of the m/z spectra, corresponding to charge 20+.


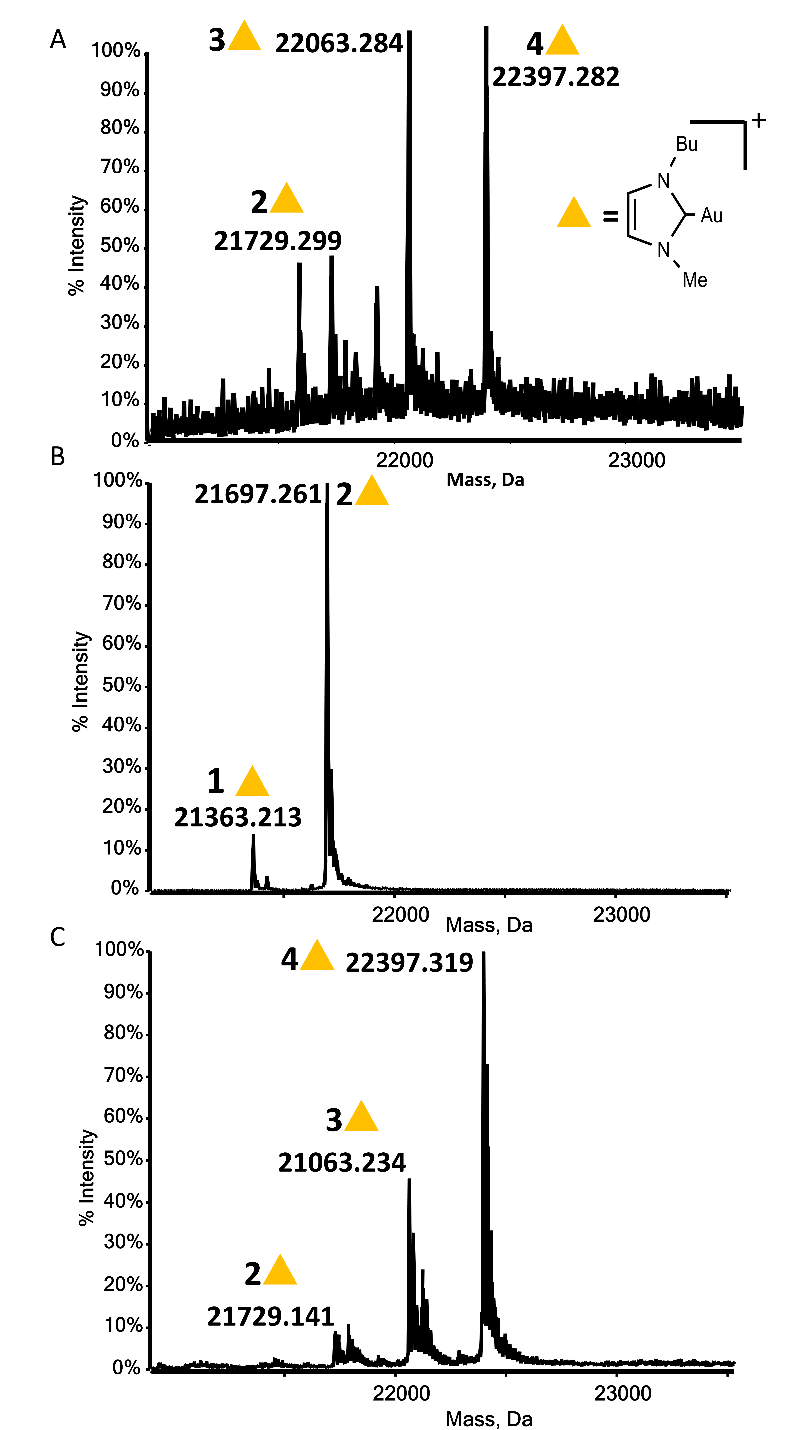


**Supplementary information Figure 2.** ESI MS spectra of the Cys🡪Ala variants of HuHf. Deconvoluted ESI-MS mass spectra of HuHf mutants 5 ×10^-6^ M in 20 mM ammonium acetate solution, incubated at 37°C for 3 hours with an added Au(NHC)Cl per ferritin subunit ratio of 5; 1% v/v of formic acid was added just before injection. From A to C: C130A, C90AC102A, and C90A. The numbers of Au(NHC)^+^ ions bound to HuHf subunits are indicated by 1-4.


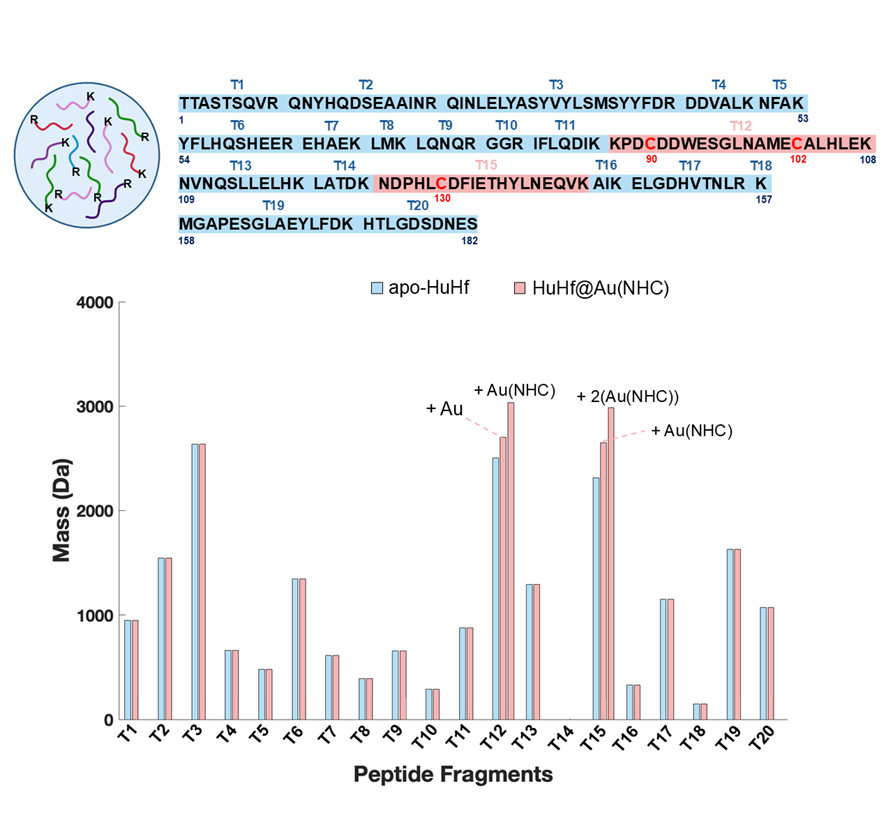


**Supplementary information Figure 3.** Identification of Au-binding peptides. Upper panel: representation of the tryptic fragments of HuHf (T1-T20); lower panel: results from the ESI-MS analysis of the tryptic fragments of apo WT HuHf (light blue) and of the NHC bioconjugates with WT HuHf (light pink). For HuHf@Au(NHC) peptides multiple species are present indicating the binding of free Au, a single molecules of Au(NHC) or two Au(NHC).


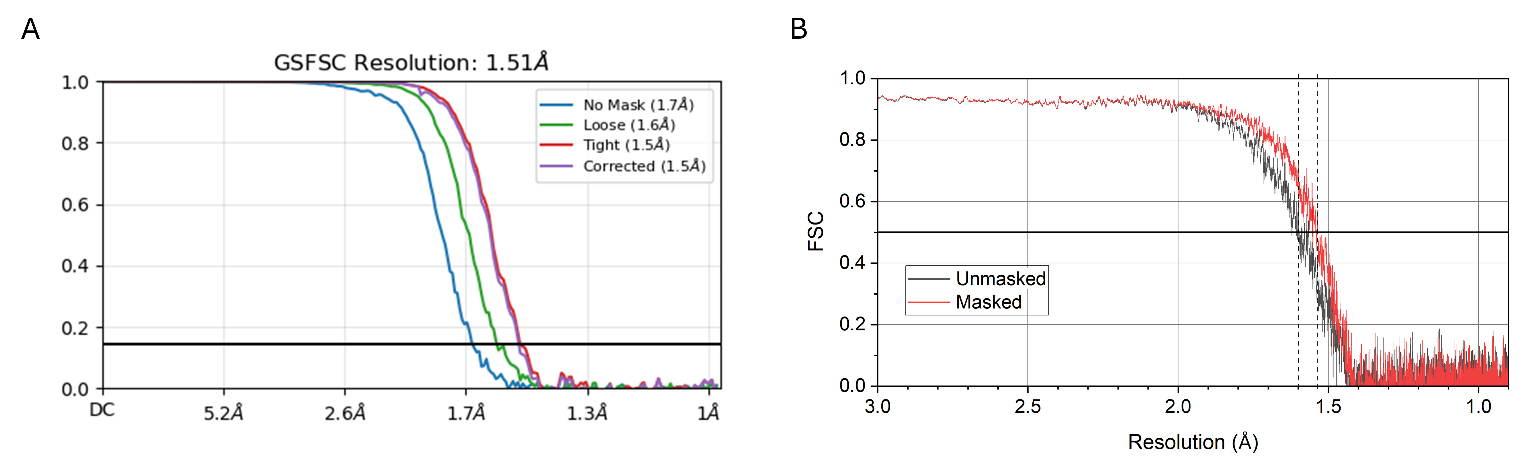


**Supplementary information Figure 4.** A) Gold-standard Fourier Shell Correlation plot for the cryo-EM data of the HuHf@Au(NHC) adduct. The plot indicates a resolution of approximately 1.5 Å. B) Model-to-map FSC curves with and without mask calculated using Phenix.^[7]^ The resolution at FSC= 0.5 is 1.56 and 1.62 Å, respectively.


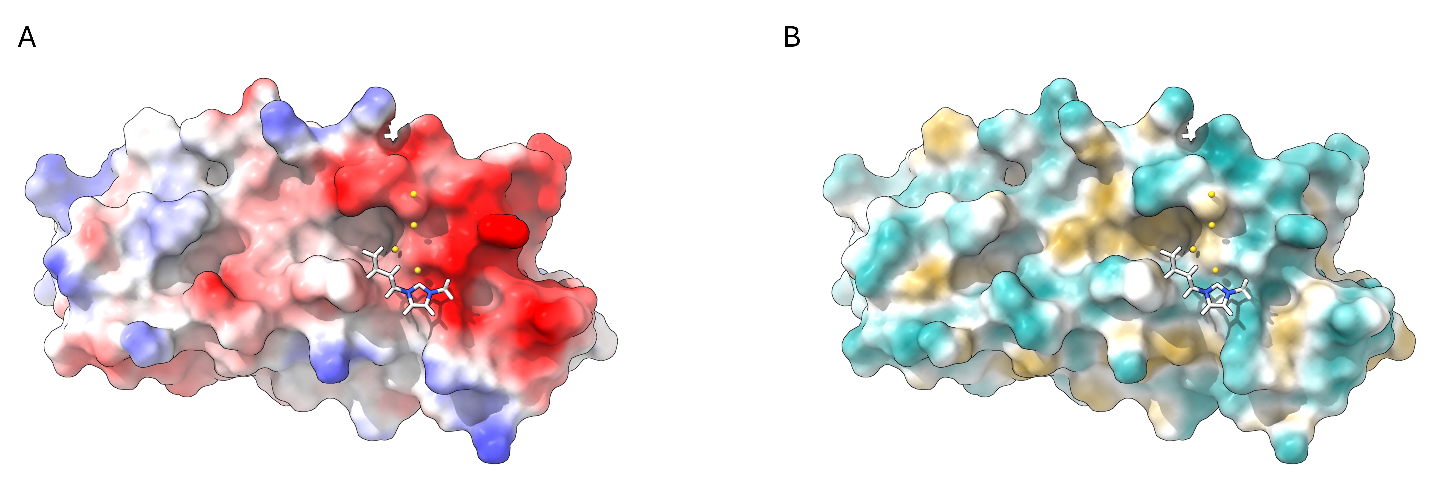
**Supplementary information Figure 5.** The surface of a single subunit of the HuHf@Au(NHC) adduct colored according to the electrostatic potential (A) or the hydrophobicity profile (B). The coloring scheme is as follows: left) from red for the regions with negative potential through white to blue for the regions with positive potential; right) from dark cyan for the most hydrophilic regions through white to dark goldenrod for most the hydrophobic regions. The gold(I) ions are shown as tallow spheres; the NHC ligand is shown using a stick representation.

**Supplementary information Table 1.** Theoretical and experimental masses for HuHf and HuHf@Au(NHC) adducts of Figure 1.

|  | **Theoretical Mass (Da)** | **Experimental Mass (Da)** |
| --- | --- | --- |
| **HuHf** | 21094.484 | 21094.311 |
| **HuHf + Au(NHC)^+^** | 21428.650 | 21428.380 |
| **HuHf + 2 Au(NHC)^+^** | 21762.827 | 21762.454 |
| **HuHf + 3 Au(NHC)^+^** | 22096.990 | 22096.562 |
| **HuHf + 3 Au(NHC)^+^ + Au** | 22292.957 | 22292.426 |
| **HuHf + 4 Au(NHC)^+^** | 22431.370 | 22431.590 |
| **HuHf + 4 Au(NHC)^+^ + Au** | 22627.729 | 22627.506 |
| **HuHf + 5 Au(NHC)^+^** | 22765.341 | 22765.683 |
| **HuHf + 6 Au(NHC)^+^** | 23099.513 | 23099.782 |

**Supplementary information Table 2.** Cryo-EM data collection and refinement statistics

| **Data collection and processing** | |
| --- | --- |
| Magnification | 165000 |
| Voltage (kV) | 300 |
| Electron exposure (e⁻/Å²) | 50 |
| Defocus range (µm) | -0.4 to -1.8 |
| Pixel size (Å²) | 0.508 |
| Symmetry | O |
| Initial particle images | 3183722 |
| Final particle images | 2736819 |
| Map resolution (Å) | 1.51 |
| FSCC threshold | 0.143 |
|  |  |
| **Refinement** | |
| Initial model used (PDB ID) | 4Y08 |
| Initial model resolution (Å) | 1.34 |
|  |  |
| **Final structure** | |
| PDB ID | 9HQ6 |
| Average B-factor (protein; Å²) | 21.32 |
| Clash score | 2.44 |
| Poor Rotamers (%) | 1.27 |
|  |  |
| *RMS Deviations* |  |
| Bond lengths (Å) | 0.002 |
| Bond Angles (°) | 0.416 |
|  |  |
| *Ramachandran plot* |  |
| Favored (%) | 97.7 |
| Allowed (%) | 2.3 |
| Outliers (%) | 0.0 |

**References**

[1] L. Messori, L. Marchetti, L. Massai, F. Scaletti, A. Guerri, I. Landini, S. Nobili, G. Perrone, E. Mini, P. Leoni, M. Pasquali, C. Gabbiani, *Inorg. Chem.* **2014**, *53*, 2396–2403.

[2] C. Pozzi, F. Di Pisa, C. Bernacchioni, S. Ciambellotti, P. Turano, S. Mangani, *Acta Cryst D* **2015**, *71*, 1909–1920.

[3] L. Cosottini, L. Massai, V. Ghini, S. Zineddu, A. Geri, M. Mannelli, S. Ciambellotti, M. Severi, T. Gamberi, L. Messori, P. Turano, *Journal of Drug Delivery Science and Technology* **2023**, *87*, 104822.

[4] E. C. Meng, T. D. Goddard, E. F. Pettersen, G. S. Couch, Z. J. Pearson, J. H. Morris, T. E. Ferrin, *Protein Science* **2023**, *32*, e4792.

[5] Brady Johnston, Johannes Elferich, Russell B. Davidson, Yuxuan Zhuang, Yinying Yao, Thibault Tubiana, Patrick Kunzmann, Rich, Olivier Laprevote, TheJeran, ludovic autin, JCZwiggelaar, Domenico Marson, Kai Niklas Spauszus, Brener Ramos, James Hooker, Jessica A. Nash, Joyce Kim, Louis Colson, Hampton Copeland, Marcelo C. R. Melo, **2024**, DOI 10.5281/ZENODO.14241983.

[6] L. Willard, *Nucleic Acids Research* **2003**, *31*, 3316–3319.

[7] D. Liebschner, P. V. Afonine, M. L. Baker, G. Bunkóczi, V. B. Chen, T. I. Croll, B. Hintze, L.-W. Hung, S. Jain, A. J. McCoy, N. W. Moriarty, R. D. Oeffner, B. K. Poon, M. G. Prisant, R. J. Read, J. S. Richardson, D. C. Richardson, M. D. Sammito, O. V. Sobolev, D. H. Stockwell, T. C. Terwilliger, A. G. Urzhumtsev, L. L. Videau, C. J. Williams, P. D. Adams, *Acta Crystallogr D Struct Biol* **2019**, *75*, 861–877.
